# Supplementary material for: 2′-O-Methyl-guanosine RNA fragments antagonize TLR7 and TLR8 to limit autoimmunity
Source: Nat Immunol. 2026 Feb 10;27(4):762–75. doi: 10.1038/s41590-026-02429-2 (PMC13043311; doi:10.1038/s41590-026-02429-2)
Supplement: Supplementary file 1 — Supplementary Fig. S1, Supplementary Tables 3–7, Supplementary methods. [file 41590_2026_2429_MOESM1_ESM.pdf]

nature immunology

Supplementary information https://doi.org/10.1038/s41590-026-02429-2

2′-*O*-Methyl-guanosine RNA fragments antagonize TLR7 and TLR8 to limit autoimmunity

In the format provided by the authors and unedited

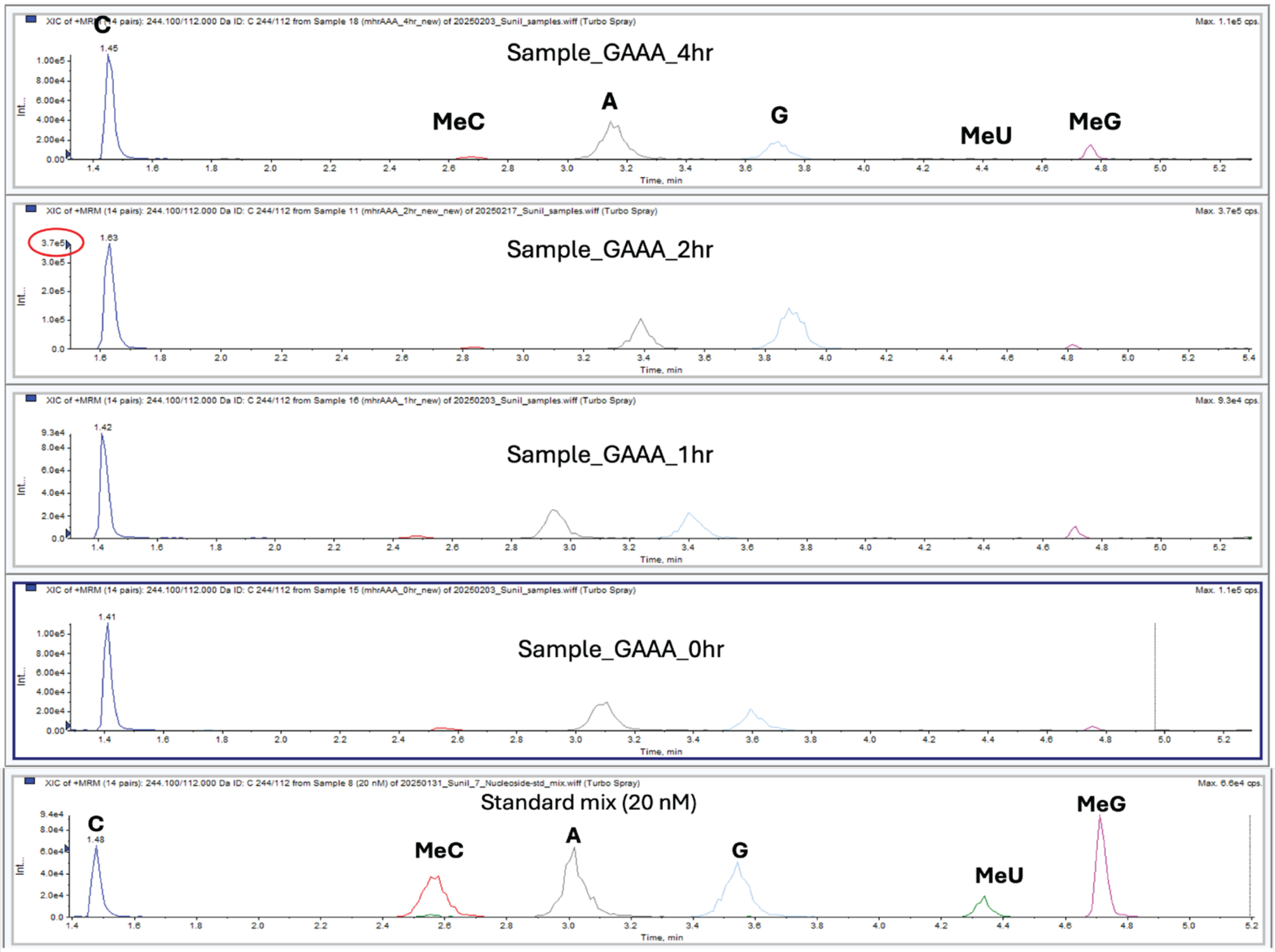

Supplementary Figure S1: LC-MS analyses of cell lysates. Overlayed Chromatogram of nucleosides and derivatives in standard solution & mGrArArA transfected samples (collected after 0, 1, 2 and 4 h) using LC-MS/MS. Nucleosides and derivatives were detected by multiple reaction monitoring (MRM) mode. MRM transitions (precursor → product ion) were as follows: G (284 → 152), C (244 → 112), A (268 → 136), MeG (243.3 → 127.2), MeC (258 → 112) and MeU (259 → 113). Chromatogram was visualised in Analyst 1.6 software (Sciex). X-axis indicates retention time; y-axis indicates intensities of MRM transitions.

SUPPLEMENTARY TABLES

Supplementary Table S3: SPR analyses.

(A) Affinity (K_D) values for SPR runs with TLR7 and oligonucleotides (Fig. 3 and S3)

	Affinity Values (µM)				
	Run 1	Run 2	Run 3	Run 4	Run 5
GUC_V1	7 ± 2	7 ± 1	6 ± 1	4 ± 2	4 ± 2
GAG_V1	38 ± 2	32 ± 2	30 ± 1	20 ± 11	11 ± 1
GUC-V6	Negligible binding observed				
GCC-V4	Negligible binding observed				

(B) Affinity (K_D) values for SPR runs with TLR8 and oligonucleotides (Fig. 3 and S3)

	Affinity Values (µM)					
	Run 1	Run 2	Run 3	Run 4	Run 5	Run 6
GAG_V1	3 ± 1	4 ± 1	5 ± 1	3 ± 0.4	4 ± 1	4 ± 1
GCC-V4	5 ± 1	7 ± 1	5 ± 1	12 ± 3	7 ± 1	11 ± 2
GUC-V6	Negligible binding observed					
GUC_V1	Not detected					

(C) Affinity (K_D) values for SPR runs with mutant TLR7 and mGrUrC^{PS} or R848 (Fig. 6)

**mGrUrC^{PS}**	Affinity Values (µM)		
TLR7 WT	0.9 ± 0.3	2 ± 0.5	1.4 ± 0.6
TLR7-T406S	0.5 ± 0.09	0.6 ± 0.1	0.6 ± 0.1
TLR7-P435S	5 ± 0.6	6 ± 0.7	6 ± 0.7
TLR7-F506L	4 ± 0.5	5 ± 0.6	5 ± 2
TLR7-F507L	16 ± 2	18 ± 2	20 ± 6
TLR7-F507S	36 ± 1	46 ± 2	42 ± 6
TLR7-S530A	0.1 ± 0.02	0.2 ± 0.04	0.2 ± 0.06
**R848**	Affinity Values (µM)		
TLR7 WT	9 ± 1	6 ± 1	7 ± 1
TLR7-T406S	5 ± 0.8	4 ± 0.7	6 ± 1
TLR7-P435A	3 ± 0.6	3.2 ± 0.8	4 ± 0.8
TLR7-F506L	9 ± 1	8 ± 1	9 ± 1
TLR7-F507L	5 ± 1	6 ± 2	7 ± 1
TLR7-F507S	4.4 ± 1	5 ± 1	6 ± 1
TLR7-S530A	3 ± 1	3.2 ± 1	4 ± 0.8

(D) Affinity (K_D) values for SPR runs with mutant TLR8 and $mGrArA^{PS}$ (Fig. 6)

**mGrArA^{PS}**	Affinity Values (µM)		
TLR8 WT	1.8 ± 0.2	1.8 ± 0.2	1.5 ± 0.2
TLR8-I403S	7.6 ± 0.5	9.7 ± 0.9	7.5 ± 0.3
TLR8-F495S	No binding observed		

(E) Affinity (K_D) values for SPR runs with WT TLR7, 2′-OMe bases, $mGrUrC^{PS}$ or R848 (Fig. 7)

**Bases**	Affinity Values (µM)		
Me-G	30 ± 6	40 ± 8	33 ± 7
Me-A	Weak binding observed (K_D ~ 1 mM)		
Me-C	No binding response observed over the assayed concentration range		
Me-U	No binding response observed over the assayed concentration range		

**Controls**	Affinity Values (µM)		
mGrUrC^{PS}	1 ± 0.3	3 ± 0.9	5 ± 0.8
R848	7 ± 2	8 ± 2	6 ± 2

(F) Affinity (K_D) values for SPR runs with WT TLR8, 2′-OMe bases, $_{m}G_{r}A_{r}A^{PS}$ or R848

Bases	Affinity Values (µM)
Me-G	Weak binding response observed
Me-A	Weak binding response observed
Me-C	No binding response observed over the assayed concentration range
Me-U	Weak binding response observed

**Controls**	Affinity Values (µM)		
$_{m}G_{r}A_{r}A^{PS}$	1.4 ± 0.9	1.5 ± 0.09	1.6 ± 0.1
R848	20 ± 3	15 ± 1	23 ± 3

Supplementary Table S4. Cryo-EM data collection, refinement and validation statistics

**Data**	TLR7/GUC-v1^{PS}	TLR7/mGrArA^{PS}	TLR7/mGrUrC^{PO}
**EMDB ID**	EMD-60515	EMD-60541	EMD-63406
**PDB ID**	PDB 8ZW2 (GUC-v1^{PS}-*SS*)<br>PDB 8ZW4 (GUC-v1^{PS}-*RR*)	PDB 8ZXE (mGrArA^{PS}-*SS*)<br>PDB 8ZXF (mGrArA^{PS}-*RR*)	PDB 9LUV
**Data collection and processing**			
Microscope	Titan Krios G4	Titan Krios G4	Titan Krios G4
Voltage (kV)	300	300	300
Detector	K3	K3	K3
Magnification	105,000	105,000	105,000
Pixel size (Å)	0.83	0.83	0.83

Total electron exposure ($e^-/Å^2$) / Frames per movie	~48 / 48	~48 / 48	~58 / 48
Defocus range (μm)	-1.2 to -2.0	-1.2 to -2.0	-1.0 to -2.0
Energy filter slit width (eV)	20	20	20
Automation software	EPU	EPU	EPU
Total movie stacks	6,615	3,111	3,288
Software	RELION v4.0.1	cryoSPARC v4.0.3	cryoSPARC v4.4.0
Initial particle images (no.)	~7,993k	~7,065k	~4,846k
Final particle images (no.)	173,248	1,446,780	197,674
Symmetry imposed	*C*2	*C*2	*C*2
Estimated accuracy angles (º) / offsets (Å) (RELION)	0.73 / 0.38	not applicable	not applicable
Map resolution (Å) (corrected/masked/unmasked, 0.143 FSC)	3.0 / 2.9 / 3.3	2.7 / 2.6 / 3.0	2.9 / 2.8 / 3.3
Map resolution (Å) (corrected/masked/unmasked, 0.5 FSC)	3.2 / 3.2 / 3.7	2.8 / 2.8 / 3.2	3.2 / 3.1 / 3.6
Local resolution range	2.8-9.5	2.7-7.2	2.7-8.4
3D FSC Sphericity value	0.979	0.986	0.983
Map sharpening B factor ($Å^2$)	-99	-50	-127
**Refinement**			
Software	Chimera, COOT, Jligand, Phenix	Chimera, COOT, Jligand, Phenix	Chimera, COOT, Phenix
Model resolution (Å) (FSC threshold = 0.5)	3.4 / 3.5	3.3 / 3.3	3.2
Model composition			
Protein chains	2 / 2	2 / 2	2
Ligands	2×GUC-v1^{PS}-*SS* or GUC-v1^{PS}-*RR*	2×mGrArA^{PS}-*SS* or mGrArA^{PS}-*RR*	2×mGrUrC^{PO}
Residues	1534 / 1534	1526 / 1526	1510
Average B-factors ($Å^2$)	127.2 / 131.9	124.6 / 124.6	145.8
R.m.s deviations			
Bond lengths (Å)	0.003 / 0.003	0.005 / 0.005	0.001
Bond angles (º)	0.540 / 0.514	1.015 / 1.015	0.360
**Validation**			
Molprobity score	1.74 / 1.86	1.64 / 1.64	1.62
Clashscore	5.68 / 5.68	5.32 / 5.32	5.56
CC (volume)	0.83 / 0.82	0.80 / 0.80	0.85
CC (mask)	0.82 / 0.81	0.79 / 0.79	0.85
CaBLAM outliers (%)	2.25 / 2.25	2.13 / 2.13	1.62
EMRinger score	2.61 / 2.64	2.89 / 2.89	2.91
Qscore	0.4990 / 0.5020	0.5130 / 0.5130	0.4870
Poor rotamers (%)	1.0 / 1.4	0.7 / 0.7	0.3
Ramachandran plot			
Favored (%)	93.5 / 93.0	94.9 / 94.9	95.5
Allowed (%)	6.1 / 6.6	4.8 / 4.8	4.5
Outliers (%)	0.4 / 0.4	0.4 / 0.4	0.0

Supplementary Table S5. Fitted pKa values of all titratable residues (D: Aspartate, E: Glutamate, H: Histidine) using GUC-v1^{PO} binding inactive TLR7 dimer structure from constant pH MD titration experiments with a range of pH from 2.0 to 10.5.

	resid	pka
D	37	<2
D	41	4.6
D	50	2.9
D	53	3.2
D	79	4.5
D	89	4.6
D	95	5.2
D	135	<2
D	198	5
D	213	6.1
D	243	4.2
D	244	5.3
D	255	<2
D	287	<2
D	320	<2
D	332	2.3
D	346	2.2
D	403	<2
D	427	<2
D	469	3.6
D	499	>10.5
D	512	<2
D	548	<2
D	555	>10.5
D	572	5.7
D	605	4.2
D	607	5.9
D	632	2.8
D	639	2.3
D	656	<2
D	671	3.4
D	705	5.5
D	745	4.6
D	753	<2
D	790	2.8
D	811	2.9
D	829	4.8

	resid	pka
E	58	4
E	93	<2
E	141	3.7
E	156	3.5
E	166	3.9
E	170	8
E	175	4.5
E	196	<2
E	229	4.5
E	242	3.8
E	291	3.9
E	318	4.3
E	329	<2
E	352	<2
E	384	3.2
E	400	3.4
E	461	<2
E	537	10.1
E	543	3.1
E	563	3.6
E	564	6.5
E	569	10.4
E	583	2.3
E	617	3.5
E	619	2.6
E	625	3.6
E	637	5.1
E	653	4.2
E	654	2.6
E	702	5.1
E	716	3
E	769	3.4
E	802	4

	resid	pka
H	46	4.6
H	55	8
H	76	6
H	86	5.5
H	90	6.2
H	298	6.7
H	304	7.1
H	337	5.7
H	394	5.3
H	464	5.3
H	515	4.2
H	558	6.3
H	566	6.9
H	578	7.1
H	587	8.6
H	630	5.1
H	708	3
H	781	6.5
H	782	7.4
H	800	5.4

Supplementary Table S6. Oligonucleotide sequences.
2′-MOE is moX, 2′-OMe is mX, DNA is dX, RNA is rX, LNA is lX, and phosphorothioate inter-nucleotide linkages are denoted with a *.

ASO #1	mA*mU*mG*mG*mC*dC*dT*dT*dT*dC*dC*dG*dT*dG*dC*mC*mA*mA*mG*mG
ASO #1-UC	mU*mC*mA*mU*mG*mG*mC*dC*dT*dT*dT*dC*dC*dG*dT*dG*dC*mC*mA*mA*mG*mG
ASO #1-GC	mG*mC*mA*mU*mG*mG*mC*dC*dT*dT*dT*dC*dC*dG*dT*dG*dC*mC*mA*mA*mG*mG
ASO #2-LNA	lC*lG*lG*dC*dC*dT*dC*dG*dG*dA*dA*dG*dC*lT*lC*lT
ASO #2-LNA-UC	mU*mC*lC*lG*lG*dC*dC*dT*dC*dG*dG*dA*dA*dG*dC*lT*lC*lT
ASO #660	mC*mU*mU*mC*mG*dT*dG*dG*dG*dG*dT*dC*dC*dT*dT*mU*mU*mC*mA*mC
ASO #660-Mut1	mG*mA*mA*mC*mG*dT*dG*dG*dG*dG*dT*dC*dC*dT*dT*mU*mU*mC*mA*mC
ASO #660-Mod	mC*mU*mU*lC*lG*dT*dG*dG*dG*dG*dT*dC*dC*dT*dT*mU*mU*mC*mA*mC
ASO #660-LNA	lT*lC*lG*dT*dG*dG*dG*dG*dT*dC*dC*T*T*lT*lT*lC
ASO #660-MOE	moC*moT*moT*moC*moG*dT*dG*dG*dG*dG*dT*dC*dC*dT*dT*moT*moT*moC*moA*moC
#660-5	mC*mU*mU*mC*mG
#660-7	mC*mU*mU*mC*mG*dT*dG
#660-9	mC*mU*mU*mC*mG*dT*dG*dG*dG
dC	mG*mG*mU*dA*dT*dC*dC*dC*dC*dC*dC*dC*dC*dC*dC*dC*dC*dC*dC*dC
dC2	mG*mG*mU*dA*dC*dC*dC*dC*dC*dC*dC*dC*dC*dC*dC*dC*dC*dC*dC*dC
dC2-MOE	moG*moG*moT*dA*dC*dC*dC*dC*dC*dC*dC*dC*dC*dC*dC*dC*dC*dC*dC*dC
dC2-LNA	lG*lG*lT*dA*dC*dC*dC*dC*dC*dC*dC*dC*dC*dC*dC*dC*dC*dC*dC*dC
5-dC	mG*mG*mU*dA*dT
ssRNA40^{PS}	rG*rC*rC*rC*rG*rU*rC*rU*rG*rU*rU*rG*rU*rG*rU*rG*rA*rC*rU*rC
ssRNA40^{PO}	rGrCrCrCrGrUrCrUrGrUrUrGrUrGrUrGrArC rUrC
GAG-v10	mG*mA*dG*dC*dC*dC*dC
B-406-AS1	rUrArArUrUrGrGrCrGrUrCrUrGrGrCrCrUrUrCrUrU
RNA9.2^{PS}[1, 2] based on RNA9.2s-DR[3]	rU*rG*rU*rC*rC*rU*rU*rC*rA*rA*rU*rG*rU*rC*rC*rU*rU*rC*rA*rA
ODN1826	dT*dC*dC*dA*dT*dG*dA*dC*dG*dT*dT*dC*dC*dT*dG*dA*dC*dG*dT*dT
ODN2216	dG*dGdG dGdGdA dCdGdA dTdCdG dTdC*dG* dG*dG*dG* dG*dG
RNA9.2s^{PO}[3]	rArGrCrUrUrArArCrCrUrGrUrCrCrUrUrCrArA
Dharmacon siGENOME Non-Targeting siRNA D-001210-02-05	rUrArArGrGrCrUrArUrGrArArGrArGrArUrArC
Dharmacon siGENOME siFBL SMARTpool M-011269-00-0005	rGrGrArArUrCrArGrUrUrArUrGrGrArGrArG<br>rGrCrCrGrUrGrArCrCrUrCrArUrUrArArCrUrU<br>rGrGrUrCrGrArGrGrCrGrGrArGrGrCrUrUrUrA<br>rGrCrGrUrArArUrGrGrArGrGrArCrArCrUrUrU

Supplementary Table S7. Multiple-reaction monitoring (MRM) parameters for nucleosides and their derivatives

Target analytes	Precursor ion (m/z)	Quantitative product ion (m/z)	CE (v)	Retention time (min)
Adenine	268	136	35	3.1
Uracil	245	113	28	2.2
Guanine	284	152	25	3.7
Cytosine	244	112	25	1.4
Methyladenine	282	136	25	4.6
Methyluracil	259	113	28	4.3
MethylGuanine	298	152	25	4.7
Methylcytosine	258	112	25	2.6

SUPPLEMENTARY METHODS

RNA sequencing. Libraries were generated using an in-house multiplex RNA-seq method (version 01/09/2021; Hudson Genomics Facility) adapted from[4]. Briefly 25 ng of total RNA from each sample was tagged with an 8 bp sample index and 10 bp unique molecular identifier (UMI) during initial poly(A) priming with the addition of a template-switching oligo. After cDNA amplification Illumina P5 adaptors with distinct i5 indexes were added by tagmentation by Nextera transposase and PCR. The final libraries were pooled based upon size-adjusted qPCR and single-end sequencing was performed on a NextSeq 2000 run using a P3 50 cycle kit (cDNA reads generated 61 nt). Base calling was performed using Dragen BCLConvert (v3.7.4).

RNA-seq analysis. RNA-seq analysis was performed in R (v4.1.0)[5]. The scPipe package (v1.14.0)[6] was employed to process and de-multiplex the data. Read alignment was performed on R1 FASTQ files using the Rsubread package (v2.6.1)[7]. An index was built using the Ensembl *Mus musculus* GRCm39 primary assembly genome file and alignment was performed with default settings. Aligned reads were mapped to exons using the sc_exon_mapping function with the Ensembl *Mus musculus* GRCm39 v104 GFF3 genome annotation file. The resulting BAM file was de-multiplexed and reads mapping to exons were associated with each individual sample using the sc_demultiplex function, taking the UMI into account, and an overall count for each gene for each sample was generated using the sc_gene_counting function (with UMI_cor = 1). Additional gene annotation was obtained using the biomaRt package (v2.48.3)[8] and a DGEList object was created with the counts and gene annotation using the edgeR package (v3.34.0)[9]. A design matrix was constructed incorporating the treatment group and donor mouse (BMDMs from n=3 mice were used). Lowly expressed genes were removed using the filterByExpr function and normalisation factors were calculated using the TMM method[9]. Counts were transformed for differential gene expression analysis using the voom method[10] and a linear model was fit using the edgeR voomLmFit function. Comparisons were made for each treatment group compared to non-treated controls using the contrasts.fit function and empirical Bayes

moderated t-statistics were calculated using the eBayes function[11]. Differentially expressed genes were determined using a false discovery rate (FDR) adjusted p value < 0.05. Volcano plots were made using the log2 fold changes of all genes versus the -log10 p value across all samples as calculated during this step.

The original multiplexed R1 FASTQ files were deposited along with the UMI counts in the NCBI Gene Expression Omnibus (GEO) with accession GSE291606.

Recombinant TLR7 and TLR8 proteins. Recombinant TLR7 and TLR8 extracellular domains were prepared as per[12, 13]. For TLR8, *Homo sapiens* TLR8 extracellular domain fused to a C-terminal thrombin-cleavage sequence followed by a protein A tag was constructed into the pMT-BiP-V5-His vector (Thermo Fisher Scientific). For TLR7, *Macaca mulatta* TLR7 extracellular domain (N167Q, N399Q, N488Q, N799Q mutations, and residues 440-445 (SEVGFC) replaced by a thrombin-cleavage sequence (LVPRGS)) was constructed into the pMT-BiP-V5-His vector. TLR7 and TLR8 antagonist binding site mutant constructs were generated via site-directed mutagenesis. For expression, stably transfected *Drosophila* S2 cells (Thermo Fisher Scientific) were cultured in EXPRESS FIVE SFM medium (Thermo Fisher Scientific). Protein expression was induced by addition of 0.5 mM copper(II) sulfate. Secreted proteins were purified using IgG Sepharose 6 Fast Flow affinity resin (Cytiva) and further subjected to thrombin cleavage and gel-filtration chromatography. Purified proteins were used for SPR and cryo-EM experiments.

Surface plasmon resonance. Using a Biacore T200 (Cytiva), TLR7 proteins were immobilised onto a Series S sensor Chip CM5 (GE) by amine coupling to a level of between ~7,500-8,000 RU for TLR7 and mutants and ~11,000-13,000 RU for TLR8 and mutants, respectively, as per the manufacturer's instructions. Briefly, at 25°C, with a flowrate of 10 µl/min, flow cells were activated with injection of 0.2 M EDC + 0.05 M NHS for 420 s. TLR7 proteins (300 µg/ml in 10 mM acetate pH 4) were coupled to the chip for 650 s at 2 ul/min), and TLR8 proteins (150 ug/ml in 10 mM

Acetate pH 4) were coupled to the chip for 600 s at 2 ul/min. Unreacted NHS was blocked on all flow cells with an injection of 1 M ethanolamine-HCl pH 8.5 for 420 s. One flow cell was used as the reference, activated and blocked as described above. The immobilisation buffer was 20 mM HEPES, 150 mM NaCl, pH 7.5.

The SPR run was performed at 20ºC with 10 mM Mes, 150 mM NaCl, pH 5.5 as the running buffer. All oligonucleotides were solubilised in running buffer, and concentration confirmed via the calculated extinction coefficient at A260 nm as well as 1D ^{1}H NMR (for R848 only). A 6-point dilution series at the given concentration range was performed for all oligonucleotides. Samples were injected for 120 s with a dissociation time of 300 s at a flow rate of 40 µl/min. Surface regeneration was performed by 2 x 60 s injections of 2 M NaCl at 30 µl/min. Data were analysed using Biacore T200 Evaluation Software (3.2). Affinity constants were determined by fitting the data using affinity analysis with a standard 1:1 Langmuir binding model. R848 affinity binding or Circular Dichroism (CD) spectroscopy were used to confirm correct folding of all TLR7 and TLR8 mutant proteins, respectively.

Cryo-EM analysis, data processing and model building. Purified recombinant TLR7 protein (0.2 mg/ml) was mixed with GUC-v1^{PS} (mixture of RR, RS, SR, and SS configurations, 0.17 mg/ml) (WuXi AppTec) or $_{m}G_{r}A_{r}A^{PS}$ (mixture of RR, RS, SR, and SS configurations, 0.3 mg/ml) (WuXi AppTec) or $_{m}G_{r}U_{r}C^{PO}$ (0.17 mg/ml) (WuXi AppTec) using a dilution buffer containing 20 mM sodium citrate tribasic dihydrate, pH 5.0 and 150 mM NaCl. For each sample, a 3-µl aliquot was applied onto a glow-discharged QUANTIFOIL® R 1.2/1.3 on Cu 300 mesh grid + 2 nm C Holey Carbon Films with ~ 2 nm continuous carbon on top (QUANTIFOIL, #C2-C14nCu30-50). For glow discharge, the grids were processed using a PIB-10 device (Vacuum Device Inc., Ibaraki, Japan) at the 'SOFT' mode for 1.2 min, which glow discharge condition was 10 Pa, 5~10 mA, 12 s according to the manufacturer's manual. The grids were blotted with 595 filter paper (TED PELLA, INC.) for 2.0 s in 100% humidity at 6°C and plunged into liquid ethane using a VitrobotMkIV (Thermo Fisher

Scientific). Cryo-EM movies were recorded by using a Titan Krios G4 microscope (Thermo Fisher Scientific) running at 300 kV and equipped with a Gatan Quantum-LS Energy Filter (GIF, 20 eV zero-loss energy filtration) and a Gatan K3 camera in the electron counting mode at the Cryo-EM facility in the University of Tokyo (Tokyo, Japan). Imaging was performed at a nominal magnification of 105,000×, corresponding to a calibrated pixel size of 0.83 Å/pixel. For the TLR7/GUC-v1^{PS} and TLR7/$_{m}G_{r}A_{r}A^{PS}$ datasets, each movie was recorded for 1.6 s and subdivide into 48 frames with a total electron exposure of about 48 $e^{-}/Å^{2}$. For the TLR7/$_{m}G_{r}U_{r}C^{PO}$ dataset, each movie was recorded at CDS mode for 2.3 s and subdivided into 48 frames with a total electron exposure of about 58 $e^{-}/Å^{2}$. EPU software (Thermo Fisher Scientific) operated at the fast acquisition mode was used for data collection.

For processing the TLR7/GUC-v1^{PS} dataset in RELION v4.0.1[14, 15], 6615 raw movie stacks were motion-corrected using MotionCor2 in RELION's own implementation[16]. The CTF parameters were determined using the CTFFIND4 program[17]. Particles were picked using the auto-picking program. Multiple rounds of 2D classification, 3D classification, 3D auto-refine, Post-processing, CTF refinement and Bayesian polishing were performed to select particle stacks (173,248 particles) for the final 3D auto-refine, which yielded an overall resolution of 3.0 Å. For processing the TLR7/mGrArA^{PS} dataset in cryoSPARC v4.0.3[18], 3111 raw movie stacks were motion-corrected using the patch motion correction, and the CTF parameters were determined using the patch CTF estimation. Particles were picked using the blob picker. Multiple rounds of 2D classification, ab initio reconstruction, heterogeneous refinement and non-uniform (NU) refinement[19] were performed to select particle stacks (1,446,780 particles) for final 3D refinement using NU refinement, which yielded an overall resolution of 2.7 Å. The TLR7/$_{m}G_{r}U_{r}C^{PO}$ dataset containing 3,288 raw movie stacks was processed in cryoSPARC v4.4.0. Initially, particles picked with blob picker were subjected to multiple rounds of 2D classification and ab initio reconstruction for generating 3D volumes for heterogeneous refinement jobs. All extracted particles from blob picker were then subjected to one round of heterogeneous refinement for particle cleaning. Multiple rounds of ab initio reconstruction,

heterogeneous refinement, 2D classification and NU refinement were performed to select particle stacks (197,674 particles) for final 3D refinement using NU refinement, which yielded an overall resolution of 2.9 Å. To obtain sharpened 3D maps, negative *B*-factors (automatically or manually determined) were applied to the 3D maps from the consensus refinement. Local resolutions of the 3D maps were estimated[20]. The final 3D maps were validated using the remote 3DFSC processing server (https://3dfsc.salk.edu/)[21]. More detailed cryo-EM image processing is provided in Extended Data Figs. 8-10.

For model building, the TLR7/Cpd-7 structure (PDB 6LW1) was used as the initial model by fitting into the cryo-EM maps using the fit in map program in Chimera software[22]. Ligand restraints (CIF files) for GUC-v1^{PS} and mGrArA^{PS} were generated using the Jligand software. The iterative cycles of manual model building in COOT[23] and real-space refinement in Phenix software[24, 25] were continued until the structures converged with reasonable geometric parameters and map-to-model fit. Both the unsharpened and the *B*-factor sharpened 3D maps were referred to during refinement in COOT. For real-space refinement in Phenix, the unsharpened 3D maps, default settings and non-default weight (0.01 or 0.02), rotamers (fit outliers) were used. All models were validated using comprehensive validation (cryo-EM) and EMRinger[26] programs in phenix. The unsharpened 3D maps were selected as the main maps and have been deposited in the Electron Microscopy Data Bank. The *B*-factor sharpened maps were also deposited as additional maps. The atomic coordinates have been deposited in the Protein Data Bank. Statistics for data collection and structural refinement are summarized in Supplementary Table S4. Structure representations were generated using ChimeraX[27]. The unsharpened 3D maps were used for structure representations.

Molecular dynamics simulations. The 2.2 Å crystal structure of monkey TLR7 in complex with imidazoquinoline (IMDQ) and GGUCCC (PDB ID: 5ZSE)[1] was used to create the homology model of wild-type human TLR7 active dimer complex using MODELLER (version 10.4)[28],and the cryo-

EM structures of GUCv-1^{PS} and ${}_{m}G_{r}A_{r}A^{PS}$ bound complex in this study were used as the templates to create the corresponding inactive human TLR7 models. The locations of crystal water molecules were refined and added using COOT[23] and phenix[29]. Only the molecules GUCCC or GUC with clear electron density were modeled in the active complex. The N-glycosylation sites on known asparagine residues were determined using the most common types on each site after checking all available electron density maps. All simulation systems were prepared using the CHARMM-GUI Solution Builder server[30], with N- and C- terminal residues patched as acetylated N terminus and methylamidated C terminus, respectively. The 2′-OMe modifications on RNA sugar groups were added and structurally refined using IQmol. For non-free-energy-perturbation simulations, the constant pH molecular dynamics (CpHMD) were conducted at pH 5; the simulation cells comprised approximately 320,000 atoms, of which approximately 100,000 were water molecules, in a box of dimensions 15 × 15 × 15 nm^3. TIP3P water parameters were used to solvate all systems. The environmental pH value in CphMD was set at value of 5.0, and 450 buffer particles were added to tackle the net charge changes due to the possible transition of protonation states of titratable residues (aspartate, glutamate and histidine). Sufficient Na^+ and Cl^- ions were introduced by replacement of water molecules to bring the systems to an electrically neutral state at an ionic strength of 0.15 M. Specific parameters for describing the interactions involving titratable residues or buffer particles for CpHMD were taken from CHARMM36* all atom force field[31, 32]. For free-energy-perturbation calculations, the initial protein structures and hybrid topology files were obtained using pmx toolkit[33], where only the residue F507 in the wild-type structure was replaced by hybrid residue F2S or F2L after being superimposed with the pmx made structure lacking these chemical modifications. The protein topology files and force field parameter file generated by CHARMM-GUI using charmm36m all atom force field[34] were modified according to the pmx hybrid topology files to include the engineered residue F2S or F2L and define possible interactions. The engineered RNA molecules with hybrid topologies (at base or 2'-O group) were created using IQmol, the topologies were manually prepared in pmx style.

All simulations were performed using the GPU-accelerated GROMACS software package (version 2023.1 and version 2021 with CpHMD module)[35]. Cgenff program was used to parameterize the small ligand R848[36]. For CphMD, steepest descent energy minimization was conducted followed by two sequential steps of equilibration (500 ps in NVT ensemble and 500 ps in NPT ensemble) with a gradual decrease in the restraining force applied to protein atoms and ligands. The LINCS algorithm[37] was applied for resetting constraints on covalent bonds to hydrogen atoms, which allowed 2 fs time steps for MD integration during the entire simulation. The particle-mesh Ewald algorithm[38] was used for calculating electrostatic interactions within a cut-off of 12 Å, with the Verlet grid cut-off-scheme[39] applied for neighbour searching, using an update frequency of 20 and a cut-off distance of 12 Å for short-range neighbours. A 12 Å cut-off was applied to account for van der Waals interactions, using a smooth switching function starting at 1.0 nm. Periodic boundary conditions were utilized in all directions. During the equilibration stages, the temperature was maintained at 303.15 K using a Berendsen-thermostat[40] with a time constant of 1.0 ps. Protein with ligands and ion-water groups including buffer particles were treated independently to increase accuracy. The pressure was maintained at 1.0 bar by isotropic application of a Berendsen-barostat[40], with a time constant of 5.0 ps. During production of molecular dynamics, the temperature was maintained at 303.15 K using a v-rescale-thermostat[41] with a time constant of 1.0 ps, and the pressure was maintained at 1.0 bar using the Parrinello–Rahman-barostat[42] isotropically, with a time constant of 5.0 ps and compressibility of 4.5×10^{-5} bar^{-1}. The active human TLR7 dimer bound with GUCCC, GUC and $_m$G$_m$U$_m$C at site 2, plus inactive human TLR7 dimer bound with GUC-v1 and its F507S mutant bound with PO-GUC-v1 were studied by running for 200 ns in 5 replicates, respectively, and a total of 5 µs long simulations were conducted. The simulations of single 2'-OMe nucleosides ($_m$G, $_m$A, $_m$C and $_m$U) at antagonist binding site were run for 400 ns in 5 replicates. The total of 13 µs long simulations were conducted.

For FEP, the Hamiltonian replica-exchange molecular dynamics (H-REMD) was conducted to improve the convergence of free energy calculations. Free energy module in GROMACS was turned on using parameter lambda to interpolate between topology A (lambda=0) to topology B (lambda=1). Soft-core potentials are used with an alpha parameter of 0.5 and sigma parameter set by 0.3 nm, and the power for lambda in the soft-core function was 1. A total of three independent steps were applied to connect the topology A (Phe) and topology B (Ser or Leu). In the first set only the partial charges on residue Phe507 were tuned by having six discrete replicas in which the lambda was 0.0, 0.2, 0.4, 0.6, 0.8 and 1.0, respectively, the residue type was still phenylalanine, and all partial charge values were zero while $lambda_{step1}$=1. In the second set, the partial charge on each atom including dummy atoms was kept zero while the vdw, mass, bonded and restraint terms of those atoms were tuned by having 16 discrete replicas in which the lambda was 0.000, 0.067, 0.134, 0.201, 0.268, 0.335, 0.402, 0.469, 0.536, 0.603, 0.670, 0.737, 0.804, 0.871, 0.938 and 1.000, respectively, and the residue could be recognized as a serine whereas all partial charge values were zero while $lambda_{step2}$=1. In the third set, only the partial charge on each atom was tuned again by having six discrete replicas in which the lambda was 0.0, 0.2, 0.4, 0.6, 0.8 and 1.0 to bring the proper partial charge back to the serine residue, an intact serine was define while $lambda_{step3}$=1. All lambda points were written out by having the calc-lambda-neighbors value of -1. Stochastic dynamics integrator was used during FEP. The replica exchange attempt was made periodically with every 1000 steps of calculation. Both apo form of inactive dimer and antagonistic RNA 3-mer (GUC-v1^{PO} or ${}_{m}G_{r}A_{r}A^{PO}$) binding dimer were used to close the thermodynamics cycle to obtain the relative binding free energy difference. Similarly, the FEP calculations for measuring relative binding free energy values between ${}_{m}G_{m}U_{d}C$ and ${}_{r}G_{r}U_{d}C$, or ${}_{m}G$ and ${}_{m}A$ at the antagonist binding site were also conducted using H-REMD following the same protocol, in which the topology A is ${}_{m}G_{m}U_{d}C$ or ${}_{m}G$, topology B is ${}_{r}G_{r}U_{d}C$ or ${}_{m}A$, and the ligand-only simulations were used for closing the thermodynamics cycle. Each set of H-REMD was repeated three times in which each replica was run for 50 ns (70 ns for ${}_{m}G$-to-${}_{m}A$), in total of 36.96 μs in FEP. Convergence of the results was assessed by plotting the free energy as a function of simulation time.

The protonated states of all aspartate, glutamate and histidine residues were pre-assigned using the results from CpHMD residue titration calculations, and using a range of pH from 2.0 to 10.5 with an interval of 0.5 (Supplementary Table S5). The system was simulated at each environmental pH for 50 ns in 5 replicates, resulting in overall simulation length of 4.5 μs.

Molecular docking. Flexible docking was conducted to investigate the potential of inhibitory RNA 3-mers to bind at the antagonist binding site in the inactive TLR8 (PDB ID: 8PFI). Autodocktools[43] was used to parameterize the RNA ligands ($_mG_mU_mC^{PO}$ and $_mG_rA_rA^{PO}$) and protein flexible and rigid components. Autodock VINA[44, 45] was used to conduct the flexible docking with exhaustiveness of 32, the grid box has a dimension of 50 Å × 50 Å × 50 Å centering at the geometry center of known antagonist (removed before docking in progress). The flexible residues chosen in TLR8 docking experiment were $F261^A$, $Y348^A$, $V378^A$, $I403^A$, $F405^A$, $E427^A$, $F494^B$, $F495^B$, $R541^B$. The inhibitory RNA binding poses with mG_1 docked at the binding pocket (if captured) were always the pose with best calculated affinity.

Co-encapsulation of GGC-v1 with Fluc mRNA in LNPs. 1 mg of CleanCap® Fluc mRNA (TriLink; L-7602) was pre-mixed by pipetting, or not, with 0.2 mg GGC-v1 (5:1 ratio by weight) before encapsulation in lipid nanoparticles (LNPs) with the composition of III-3: Iva PEG-lipid: DSPC: Cholesterol = 47.4:10:40.9:1.7 (by molar ratio) using a Nanoassemblr Ignite (PrecisionNanoSystems). Resulting LNP formulations were dialyzed against 20 mM pH 7.4 Tris buffer and diluted with PBS after dialysis. Concentrations of the LNP-formulated mRNA samples were adjusted to 0.2 g/L. LNP particle size, polydispersity index (PDI), zeta potential, and mRNA encapsulation rate (Ribogreen assay) were assessed and comparable for both formulations generated. LC-MS/MS and RT-qPCR were used to quantitate GGC-v1 and Fluc mRNA, respectively, in the LNPs. Fluc primers used were: Fluc Forward primer: 5′-CCACATCGAGGTGGACATCA-3’; Fluc Reverse primer: 5′-AGCACGGGCATGAAGAACTG-3’. The RT-qPCR analyses were performed

on a QuantStudio® 5 Real-Time PCR System (Applied Biosystems) and data analysed using QuantStudio Design and Analysis Software v1.5.2.; LC-MS/MS analyses were conducted on Waters Acquity premier UPLC system (Waters Corporation, Milford, MA) coupled QTRAP 6500 mass spectrometer (Sciex, Framingham, MA) and analysed with Analyst Software v 1.6.3.

Mouse studies.

All the animal experiments complied with relevant local ethical regulations. Mice experienced a 12 hour light/dark cycle with temperature ~22°C and humidity of ~40%. No statistical methods were used to pre-determine sample sizes but our sample sizes are similar to those reported in previous publications: Aldara model[46], R848 model[47], and mRNA/LNP model[48]. Animals were assigned to the various experimental groups randomly. Mice were fed Specialty Feeds (specialtyfeeds.com) Irradiated Rat and Mouse Cubes (#SF00-100Irr).

Aldara-driven skin inflammation model. These experiments were approved in advance by an Animal Ethics Committee at Monash Medical Centre (MMCB/2022/18 and MMCB/2023/19) and were carried out in accordance with "Australian Code of Practice for the Care and Use of Animals for Scientific Purposes. 24x Eight weeks old C57Bl/6J female mice (purchased from Monash Animal Research Platform) were used in these experiments and were housed in SPF (Specific Pathogen Free) conditions. Mice were anaesthetized for 1-2 minutes with a vapor 17 vaporizer machine (oxygen flow rate of 1-4 litres/min) with fresh oxygen and 5% isoflurane. Upon induction of anesthesia, back hair was removed using sensitive hair removal cream (Nair). A small drop of Nair cream was applied to mouse's upper back using a cotton bud and smeared to cover a 2 cm^2 patch. The Nair cream was allowed to sit for ~one minute before being wiped off in a nose-tail motion using a sterile cotton bud to remove hair from the back skin. Thereafter, mice were treated topically to the hairless back skin with 10 µl of 10-60 µg of highly pure GGC-v1 oligonucleotide (>99.4%) resuspended in sterile PBS, combined with 20 µl of ice cold 30% Pluronic F-127 gel solution in sterile PBS (Sigma), and allowed

to absorb for 1 min. 50 mg of Aldara cream (containing imiquimod, 5% w/v) was subsequently applied to the back skin using a cotton bud. One cohort of mice was treated with Vaseline cream as vehicle control for the Aldara cream. These treatments were repeated daily for 4 consecutive days. Mice were scored daily for back erythema (redness) and skin scaling[46], and scoring was blinded. Spleens and skin samples were collected at the end of the experiment (day 5).

Histology and immunofluorescence. Immunofluorescence staining was performed using the Opal staining kit (Akoya Biosciences) as per manufacturer's instruction[49]. Skin tissue sections were deparaffinised, and antigen retrieval was performed by microwave treatment. Endogenous peroxidases were quenched by treating the tissue sections with 3% H_2O_2 for 10 min. Tissue sections were incubated in blocking buffer (Akoya Biosciences) for 10 min at room temperature. After this, sections were incubated overnight with CD45 (D3F8Q) antibody (1:200 dilution in SignalStain Antibody Diluent, Cell signalling Technology, lot #4) at 4°C and incubated with secondary HRP (SignalStain Boost IHC Detection Reagent, Cell signalling) for 30 min at room temperature. The sections were incubated with Opal 570 working solution (Akoya Biosciences) for 10 min at room temperature and counterstained with DAPI (Akoya Biosciences). Slides were scanned using the VS120 Slide Scanning System (Olympus). Quantification of positive cells was done using ImageJ software (National Institutes of Health).

RNA purification from skin biopsies. Skin samples were cut into small pieces and suspended in 300 µl of lysis buffer (PureLink RNA Mini Kit – Thermo Fisher Scientific), prior to homogenisation on ice in 5-15 sec bursts for 2-5 min using IKA T10 homogeniser (T10 basic ULTRA-TURRAX). The homogenates were subsequently centrifuged at 16,000 x g for 5 minutes and the supernatants were transferred to a clean RNase-free tubes and total RNA extracted with the PureLink RNA Mini Kit (Thermo Fisher Scientific).

Systemic R848 challenge. 12x C57BL/6NCrl female mice from ANU animal services (used under Australian National University animal ethics, reference A2022/18) were injected intravenously with 200 µg of GGC-v1 conjugated with *in vivo*-jetPEI® (Polyplus #101000030) in 5% Glucose, 1 h prior to intraperitoneal injection with 25 µg R848 VacciGrade (InvivoGen #vac-r848). Two hours after injection of R848, mice were bled retroorbitally for collection of serum and sacrificed for spleen collection. The spleen was processed to a single-cell suspension and following lysis of red blood cells, total RNA was purified from $5x10^6$ total splenocytes using the Isolate II RNA mini-kit (Meridian Biosciences # BIO-52072) according to the manufacturer's instructions. RNA was transcribed into cDNA using SuperScript IV Reverse Transcriptase (Thermo Fisher Scientific #18090010) according to the manufacturer's instructions. RT-qPCR analyses were carried out with the Power SYBR Green Master Mix (Thermo Fisher Scientific) on an Applied Biosystems 7900 machine (Thermo Fisher Scientific) using 7900 SDS v2.4.1 software. Each PCR was performed in technical triplicates with mouse *Gapdh* used as the reference gene. Relative gene expression was calculated using the $2^{-\Delta\Delta Ct}$ method. The primers used for Fig. 5a were as follows: mIl10-FWD GCT CTT ACT GAC TGG CAT GAG, mIl10-REV CGC AGC TCT AGG AGC ATG TG; mTNF-FWD CGC TCT TCT GTC TAC TGA ACT TCG G, mTNF-REV AGAACTGATGAGAGGGAGGCCATTT; mIl6-FWD TCT ATA CCA CTT CAC AAG TCG GA mIl6-REV GAA TTG CCA TTG CAC AAC TCT TT; mGapdh-FWD AAT GTG TCC GTC GTG GAT, mGapdh-REV CTC AGA TGC CTG CTT CAC. Analyses of samples were conducted blinded to the group used.

Systemic Fluc mRNA administration. These animal experiments were conducted by Eurofins Discovery Pharmacology Discovery Services Taiwan, and approved by Institutional Animal Care and Use Committee reference IN020-08202020-27736. Thirteen 8-week-old 129X1/SvJ female mice (purchased from Jackson Laboratories #000691) (~25 g) were injected intravenously (i.v.) with ~20 µg Fluc mRNA (actual 20.349 µg and 20.484 µg) encapsulated in LNPs with, or without, GGC-

v1 (see details above). Bioluminescence imaging was performed at 6 h post-injection using an IVIS Spectrum®. Briefly, mice were anaesthetised with 4 % isoflurane, and 3 mg/mouse d-luciferin potassium salt in PBS was administered i.v. (in order to quantify luminescence expression using images recorded for 3 minutes, starting 5 minutes post-luciferin injection). For bioluminescence image analysis, regions of interest encompassing the area of signal were defined using the IVIS Spectrum, and the total number of photons per second [counts/second (cps)] was recorded with Living Image™ software v4.5.2. Blood was sampled at 6 h post-injection by submandibular bleed for serum quantification of IFN-α by ELISA (Invitrogen; BMS 6027 – analysed on TECAN Infinite F50 with Tecan i-control software) and other inflammatory cytokines by Bio-Plex Pro Mouse Cytokine 23-plex Assay (Biorad) on a BIO-RAD Bio-Plex 200 Luminex (using Bio-Plex Manager Software 6.0). Livers were also collected at 24 h and snap-frozen at -80°C until analysis. To measure luciferase activity, cell culture lysis reagent (CCLR, Promega) was added to fresh livers at 1 ml per 1 g of tissue, followed by homogenization with a polytron (<10 sec on ice). The samples were centrifuged at 16,000 g for 10 min at 4°C. Liver lysates were first normalized to 40 mg/mL with CCLR, then diluted 500X for luciferase activity measurement within 5 minutes. Luciferase activity was quantified as relative light units (RLU). Analyses of samples were conducted blinded to the group used.

References

1. Zhang, Z. *et al.* Structural Analyses of Toll-like Receptor 7 Reveal Detailed RNA Sequence Specificity and Recognition Mechanism of Agonistic Ligands. *Cell Rep* **25**, 3371-3381 e3375 (2018).

2. Shibata, T. *et al.* Guanosine and its modified derivatives are endogenous ligands for TLR7. *Int Immunol* **28**, 211-222 (2016).

3. Hornung, V. *et al.* Sequence-specific potent induction of IFN-alpha by short interfering RNA in plasmacytoid dendritic cells through TLR7. *Nat Med* **11**, 263-270 (2005).

4. Ullah, T.R. *et al.* Pharmacological inhibition of TBK1/IKKepsilon blunts immunopathology in a murine model of SARS-CoV-2 infection. *Nat Commun* **14**, 5666 (2023).

5. Team, R.C. R: A language and environment for statistical computing. (2021).

6. Pertea, M. *et al.* scPipe: A flexible R/Bioconductor preprocessing pipeline for single-cell RNA-sequencing data. *PLOS Computational Biology* **14** (2018).

7. Liao, Y., Smyth, G.K. & Shi, W. The R package Rsubread is easier, faster, cheaper and better for alignment and quantification of RNA sequencing reads. *Nucleic Acids Research* **47**, e47-e47 (2019).

8. Durinck, S., Spellman, P.T., Birney, E. & Huber, W. Mapping identifiers for the integration of genomic datasets with the R/Bioconductor package biomaRt. *Nature Protocols* **4**, 1184-1191 (2009).

9. Robinson, M.D., McCarthy, D.J. & Smyth, G.K. edgeR: a Bioconductor package for differential expression analysis of digital gene expression data. *Bioinformatics* **26**, 139-140 (2009).

10. Law, C.W., Chen, Y., Shi, W. & Smyth, G.K. voom: precision weights unlock linear model analysis tools for RNA-seq read counts. *Genome Biology* **15** (2014).

11. Smyth, G.K. Linear models and empirical bayes methods for assessing differential expression in microarray experiments. *Stat Appl Genet Mol Biol* **3**, Article3 (2004).

12. Zhang, Z. *et al.* Structural Analysis Reveals that Toll-like Receptor 7 Is a Dual Receptor for Guanosine and Single-Stranded RNA. *Immunity* **45**, 737-748 (2016).

13. Tanji, H., Ohto, U., Shibata, T., Miyake, K. & Shimizu, T. Structural reorganization of the Toll-like receptor 8 dimer induced by agonistic ligands. *Science* **339**, 1426-1429 (2013).

14. Scheres, S.H. RELION: implementation of a Bayesian approach to cryo-EM structure determination. *J Struct Biol* **180**, 519-530 (2012).

15. Kimanius, D., Dong, L., Sharov, G., Nakane, T. & Scheres, S.H.W. New tools for automated cryo-EM single-particle analysis in RELION-4.0. *Biochem J* **478**, 4169-4185 (2021).

16. Zheng, S.Q. *et al.* MotionCor2: anisotropic correction of beam-induced motion for improved cryo-electron microscopy. *Nat Methods* **14**, 331-332 (2017).

17. Rohou, A. & Grigorieff, N. CTFFIND4: Fast and accurate defocus estimation from electron micrographs. *J Struct Biol* **192**, 216-221 (2015).

18. Punjani, A., Rubinstein, J.L., Fleet, D.J. & Brubaker, M.A. cryoSPARC: algorithms for rapid unsupervised cryo-EM structure determination. *Nat Methods* **14**, 290-296 (2017).

19. Punjani, A., Zhang, H. & Fleet, D.J. Non-uniform refinement: adaptive regularization improves single-particle cryo-EM reconstruction. *Nat Methods* **17**, 1214-1221 (2020).

20. Cardone, G., Heymann, J.B. & Steven, A.C. One number does not fit all: mapping local variations in resolution in cryo-EM reconstructions. *J Struct Biol* **184**, 226-236 (2013).

21. Tan, Y.Z. *et al.* Addressing preferred specimen orientation in single-particle cryo-EM through tilting. *Nat Methods* **14**, 793-796 (2017).

22. Pettersen, E.F. *et al.* UCSF Chimera--a visualization system for exploratory research and analysis. *J Comput Chem* **25**, 1605-1612 (2004).

23. Emsley, P. & Cowtan, K. Coot: model-building tools for molecular graphics. *Acta Crystallogr D Biol Crystallogr* **60**, 2126-2132 (2004).

24. Adams, P.D. *et al.* PHENIX: a comprehensive Python-based system for macromolecular structure solution. *Acta Crystallogr D Biol Crystallogr* **66**, 213-221 (2010).

25. Afonine, P.V. *et al.* Real-space refinement in PHENIX for cryo-EM and crystallography. *Acta Crystallogr D Struct Biol* **74**, 531-544 (2018).

26. Barad, B.A. *et al.* EMRinger: side chain-directed model and map validation for 3D cryo-electron microscopy. *Nat Methods* **12**, 943-946 (2015).

27. Pettersen, E.F. *et al.* UCSF ChimeraX: Structure visualization for researchers, educators, and developers. *Protein Sci* **30**, 70-82 (2021).

28. Webb, B. & Sali, A. Comparative Protein Structure Modeling Using MODELLER. *Curr Protoc Bioinformatics* **54**, 5 6 1-5 6 37 (2016).

29. Adams, P.D. *et al.* PHENIX: building new software for automated crystallographic structure determination. *Acta Crystallogr D Biol Crystallogr* **58**, 1948-1954 (2002).

30. Jo, S., Kim, T., Iyer, V.G. & Im, W. CHARMM-GUI: a web-based graphical user interface for CHARMM. *J Comput Chem* **29**, 1859-1865 (2008).

31. Buslaev, P. *et al.* Best Practices in Constant pH MD Simulations: Accuracy and Sampling. *J Chem Theory Comput* **18**, 6134-6147 (2022).

32. Aho, N. *et al.* Scalable Constant pH Molecular Dynamics in GROMACS. *J Chem Theory Comput* **18**, 6148-6160 (2022).

33. Gapsys, V., Michielssens, S., Seeliger, D. & de Groot, B.L. pmx: Automated protein structure and topology generation for alchemical perturbations. *J Comput Chem* **36**, 348-354 (2015).

34. Huang, J. *et al.* CHARMM36m: an improved force field for folded and intrinsically disordered proteins. *Nat Methods* **14**, 71-73 (2017).

35. Van Der Spoel, D. *et al.* GROMACS: fast, flexible, and free. *J Comput Chem* **26**, 1701-1718 (2005).

36. Vanommeslaeghe, K. & MacKerell, A.D., Jr. Automation of the CHARMM General Force Field (CGenFF) I: bond perception and atom typing. *J Chem Inf Model* **52**, 3144-3154 (2012).

37. Hess, B., Bekker, H., Berendsen, H.J.C. & Fraaije, J.G.E.M. LINCS: A linear constraint solver for molecular simulations. *Journal of Computational Chemistry* **18**, 1463-1472 (1997).

38. Darden, T., York, D. & Pedersen, L. Particle mesh Ewald: An N·log(N) method for Ewald sums in large systems. *The Journal of Chemical Physics* **98**, 10089-10092 (1993).

39. Verlet, L. Computer "Experiments" on Classical Fluids. I. Thermodynamical Properties of Lennard-Jones Molecules. *Physical Review* **159**, 98-103 (1967).

40. Berendsen, H.J.C., Postma, J.P.M., van Gunsteren, W.F., DiNola, A. & Haak, J.R. Molecular dynamics with coupling to an external bath. *The Journal of Chemical Physics* **81**, 3684-3690 (1984).

41. Bussi, G., Donadio, D. & Parrinello, M. Canonical sampling through velocity rescaling. *The Journal of Chemical Physics* **126** (2007).

42. Parrinello, M. & Rahman, A. Polymorphic transitions in single crystals: A new molecular dynamics method. *Journal of Applied Physics* **52**, 7182-7190 (1981).

43. Morris, G.M. *et al.* AutoDock4 and AutoDockTools4: Automated docking with selective receptor flexibility. *J Comput Chem* **30**, 2785-2791 (2009).

44. Trott, O. & Olson, A.J. AutoDock Vina: improving the speed and accuracy of docking with a new scoring function, efficient optimization, and multithreading. *J Comput Chem* **31**, 455-461 (2010).

45. Eberhardt, J., Santos-Martins, D., Tillack, A.F. & Forli, S. AutoDock Vina 1.2.0: New Docking Methods, Expanded Force Field, and Python Bindings. *J Chem Inf Model* **61**, 3891-3898 (2021).

46. van der Fits, L. *et al.* Imiquimod-induced psoriasis-like skin inflammation in mice is mediated via the IL-23/IL-17 axis. *J Immunol* **182**, 5836-5845 (2009).

47. Hemmi, H. *et al.* Small anti-viral compounds activate immune cells via the TLR7 MyD88-dependent signaling pathway. *Nat Immunol* **3**, 196-200 (2002).

48. Tahtinen, S. *et al.* IL-1 and IL-1ra are key regulators of the inflammatory response to RNA vaccines. *Nat Immunol* **23**, 532-542 (2022).

49. Ying, L. *et al.* Anti-CD40L therapy prevents the formation of precursor lesions to gastric B-cell MALT lymphoma in a mouse model. *J Pathol* **259**, 402-414 (2023).
