# Supplementary material for: 2′-O-Methyl-guanosine RNA fragments antagonize TLR7 and TLR8 to limit autoimmunity
Source: Nat Immunol. 2026 Feb 10;27(4):762–75. doi: 10.1038/s41590-026-02429-2 (PMC13043311; doi:10.1038/s41590-026-02429-2)
Supplement: Supplementary file 2 — Reporting Summary [file 41590_2026_2429_MOESM2_ESM.pdf]

nature portfolio

Corresponding author(s): Michael Gantier

Last updated by author(s): Dec 19, 2025

Reporting Summary

Nature Portfolio wishes to improve the reproducibility of the work that we publish. This form provides structure for consistency and transparency in reporting. For further information on Nature Portfolio policies, see our Editorial Policies and the Editorial Policy Checklist.

Statistics

For all statistical analyses, confirm that the following items are present in the figure legend, table legend, main text, or Methods section.

n/a	Confirmed	
☐	☒	The exact sample size (*n*) for each experimental group/condition, given as a discrete number and unit of measurement
☐	☒	A statement on whether measurements were taken from distinct samples or whether the same sample was measured repeatedly
☐	☒	The statistical test(s) used AND whether they are one- or two-sided<br>*Only common tests should be described solely by name; describe more complex techniques in the Methods section.*
☒	☐	A description of all covariates tested
☐	☒	A description of any assumptions or corrections, such as tests of normality and adjustment for multiple comparisons
☐	☒	A full description of the statistical parameters including central tendency (e.g. means) or other basic estimates (e.g. regression coefficient) AND variation (e.g. standard deviation) or associated estimates of uncertainty (e.g. confidence intervals)
☐	☒	For null hypothesis testing, the test statistic (e.g. *F*, *t*, *r*) with confidence intervals, effect sizes, degrees of freedom and *P* value noted<br>*Give P values as exact values whenever suitable.*
☒	☐	For Bayesian analysis, information on the choice of priors and Markov chain Monte Carlo settings
☒	☐	For hierarchical and complex designs, identification of the appropriate level for tests and full reporting of outcomes
☒	☐	Estimates of effect sizes (e.g. Cohen's *d*, Pearson's *r*), indicating how they were calculated

Our web collection on statistics for biologists contains articles on many of the points above.

Software and code

Policy information about availability of computer code

Data collection

1. For SPR analyses, a Biacore T200 (Cytiva) was used
2. For ELISA and luminescence collection with Fluostar OPTIMA the OPTIMA-Control v2.2R2 software was used.
3. For LegendPlex TNF analyses, Sample acquisition was performed using a BD LSR-II flow cytometer (BD Biosciences)
4. LC-MS/MS analyses of 3-mer in LNP were conducted on Waters Acquity premier UPLC system (Waters Corporation, Milford, MA) coupled QTRAP 6500 mass spectrometer (Sciex, Framingham, MA)
5. For 2'OMe nucleoside detection, LC-MS/MS analyses were performed using a Shimadzu LC-30AD binary pump system coupled to a hybrid triple quadrupole/linear ion trap mass spectrometer (QTRAP 5500). Chromatographic separation was achieved using a Synergi Hydro-RP.
6. RT-qPCR were conducted on QuantStudio 6 Flex RT-PCR system (Thermo Fisher)
7. IVIS Spectrum was used for in vivo luciferase quantification
8. A Bio-Rad ChemiDoc was used for Western blot imaging
9. serum quantification of IFN-by ELISA was analysed on TECAN Infinite F50
10. BIO-RAD Bio-Plex 200 Luminex was used for 23-plex assay analyses
11. Cryo-EM movies were recorded by using a Titan Krios G4 microscope equipped with a Gatan Quantum-LS Energy Filter
12. VS120 Slide Scanning System (Olympus) for scanning histology slides
13. Analyses of PBMC CBA were conducted with Attune NxT (Thermo Fisher).
14. LNP luciferase qPCR was carried out on QuantStudio Design
15. Applied Biosystems 7900 machine (Thermo Fisher Scientific) for R848 qPCR experiments
16. Applied Biosystems QuantStudio 3 machine for RTL-P assay PCRs

Data analysis	Software<br>The software used below match the equipments above.<br>1. For SPR analyses, the Biacore T200 Evaluation Software Version 3.2 (Cytiva) was used.<br>2. MARS Data analysis software 3.01R2 (BMG Labtech) was used for luminescence and absorbance analyses.<br>3. Legendplex data were analysed with the LEGENDplex™ Data Analysis Software Suite (BioLegend)<br>4. LNP LC-MS/MS analyses of 3mers were performed on Analyst Software v 1.6.3.<br>5. LC-MS/MS Data analysis of nucleosides was conducted using MultiQuant SoftwareTM 2.0<br>6. QuantStudio™ Real-Time PCR Software v1.7.2 for qPCR analyses from QuantStudio 6 Flex<br>7. Living Image™ software v4.5.2 for IVIS analyses<br>8. For Western blot analyses, the ImageLab software v6.1 was used.<br>9. Tecan i-control software for analyses of IFN ELISA.<br>10. Bio-Plex Manager Software 6.0 was used for 23-plex analyses.<br>11. For processing the TLR7/GUC-v1PS dataset, RELION v4.0.1 was used. The CTF parameters were determined using the CTFFIND4. For processing the TLR7/mGrArAPS dataset, cryoSPARC v4.0.3 was used. The TLR7/mGrUrCPO dataset was processed in cryoSPARC v4.4.0.<br>12. IImageJ 1.53 was used to analyse histological microscopy images.<br>13. FlowJo Sowftare v10.9 (BD) was used for analyses of CBA results on Attune<br>14. Analysis Software v1.5.2 for Luciferase qPCR was used for luciferase qPCRs<br>15. 7900 SDS v2.4.1 software for R848 qPCR experiments<br>16. QuantStudio Design & Analysis Software v1.5.2<br><br>Statistical analyses were carried out using Prism 10 (GraphPad Software Inc.)<br><br>Molecular Dymanics:<br>homology model of wild-type human TLR7 active dimer complex was generated using MODELLER (version 10.4)<br>All simulations were performed using the GPU-accelerated GROMACS software package (version 2023.1 and version 2021 with CpHMD module)<br>Autodock VINA and Autodocktools were also used for docking predictions (see methods).<br><br>For RNA sequencing analyses:<br>Dragen BCLConvert (v3.7.4) for base calling<br>R (v4.1.0) data analysis was conducted with the following packages:<br>scPipe package (v1.14.0) read processing, demultiplexing, gene counting<br>Rsubread package (v2.6.1) read alignment<br>biomaRt package (v2.48.3) gene annotation<br>edgeR package (v3.34.0) count filtering, normalisation, linear model fitting

For manuscripts utilizing custom algorithms or software that are central to the research but not yet described in published literature, software must be made available to editors and reviewers. We strongly encourage code deposition in a community repository (e.g. GitHub). See the Nature Portfolio guidelines for submitting code & software for further information.

Data

Policy information about availability of data

All manuscripts must include a data availability statement. This statement should provide the following information, where applicable:

- Accession codes, unique identifiers, or web links for publicly available datasets
- A description of any restrictions on data availability
- For clinical datasets or third party data, please ensure that the statement adheres to our policy

RNA sequencing data has been deposited in the NCBI Gene Expression Omnibus (GEO) with accession GSE291606.
The cryo-EM maps have been deposited at the Electron Microscopy Data Bank under the following accession codes: EMD-60515 (TLR7/GUC-v1PS complex), EMD-60541 (TLR7/mGrArAPS complex) and EMD-63406 (TLR7/mGrUrCPO complex). The coordinates of the atomic models have been deposited at the Protein Data Bank (PDB) under the following accession codes: TLR7/GUC-v1PS-SS (8ZW2), TLR7/GUC-v1PS-RR (8ZW4), TLR7/mGrArAPS-SS (8ZXE), TLR7/mGrArAPS-RR (8ZXF) and TLR7/mGrUrCPO (9LUV).

Research involving human participants, their data, or biological material

Policy information about studies with human participants or human data. See also policy information about sex, gender (identity/presentation), and sexual orientation and race, ethnicity and racism.

Reporting on sex and gender	The PBMC experiments were conducted in 2 males and one female healthy volunteers (40-45 years old)
Reporting on race, ethnicity, or other socially relevant groupings	NA
Population characteristics	NA
Recruitment	The participants provided written informed consent before participation, using the Monash Health Human Research Ethics Committee–approved Participant Information and Consent Form (Protocol RES-18-0000-363A). No financial compensation was provided.

Ethics oversight | Monash Health Human Research Ethics Committee approval #RES-18-0000-363A

Note that full information on the approval of the study protocol must also be provided in the manuscript.

Field-specific reporting

Please select the one below that is the best fit for your research. If you are not sure, read the appropriate sections before making your selection.

☒ Life sciences ☐ Behavioural & social sciences ☐ Ecological, evolutionary & environmental sciences

For a reference copy of the document with all sections, see nature.com/documents/nr-reporting-summary-flat.pdf

Life sciences study design

All studies must disclose on these points even when the disclosure is negative.

Sample size	No statistical methods were used to pre-determine sample sizes but our sample sizes are similar to those reported in previous publications. For Aldara experiments, we used 7 mice for experimental groups and 3 mice for non-treated controls which had previously found was sufficient to achieve statistical power in aldara-treated mice (PMID: 19380832).<br>For R848 systemic injection studies in Fig 5A, we used 4/5 mice per treatment group as we anticipated it was the minimum required for these analyses to reach significance based on literature (PMID: 11812998). For LNP injections in Figure 5E/F/G, we used 5 mice per group as as we anticipated it was the minimum required to reach significance based on literature (PMID: 35332327).
Data exclusions	For Flt3L-DC data, IFNa was only significantly detected in 2 out of 3 mice (however TNFa was detected in 3/3 mice). For PBMCs, IFNg levels from one donor saturated the assay and were omitted in the calculations of the averages.
Replication	In vitro experiments were all reliably reproduced a minimum of two independent times except for the DNA trimer screens shown in Table S1 which were only conducted once due to limited activities seen. The aldara in vivo studies were independently replicated more than 2 times. Similarly the R848 systemic challenge was independently confirmed with another TLR7 inhibiting 3-base oligonucleotide. The LNP in vivo studies were only conducted once as robust significance was reached with 5 animals in each group.
Randomization	Mice were randomly allocated to their group for all in vivo studies. For preparation of bone marrow derived macrophages and DCs from Kika mutant mice or WT mice, mice from the same genotype and same age were used.
Blinding	Data collection and analysis were not performed blind to the conditions of the experiments unless otherwise stated in the methods.<br>Mice studies:<br>For Aldara studies, the treatments were not blinded but scoring of the mice was conducted blinded.<br>For analyses of sera from LNP studies, the treatment and analyses of LNP with FLuc or FLuc+GGCv1 were conducted blinded.<br>For the systemic R848 challenge experiments, the treatments were not blinded but similarly, analyses of the TNF and RTqPCRs in collected samples were conducted blinded.<br>Cells studies:<br>All oligonucleotide screens were performed blinded (with no knowledge of the sequences used).

Reporting for specific materials, systems and methods

We require information from authors about some types of materials, experimental systems and methods used in many studies. Here, indicate whether each material, system or method listed is relevant to your study. If you are not sure if a list item applies to your research, read the appropriate section before selecting a response.

Materials & experimental systems

n/a	Involved in the study	
☐	☒	Antibodies
☐	☒	Eukaryotic cell lines
☒	☐	Palaeontology and archaeology
☐	☒	Animals and other organisms
☒	☐	Clinical data
☒	☐	Dual use research of concern
☒	☐	Plants

Methods

n/a	Involved in the study	
☒	☐	ChIP-seq
☒	☐	Flow cytometry
☒	☐	MRI-based neuroimaging

Antibodies

Antibodies used	We used:<br>1. CD45 (D3F8Q) antibody (#70257S Cell signalling Technology - Lot #4) (1:200),<br>2. Anti-Fibrillarin (38F3) antibody (Abcam ab4566 Lot 1088391-1) (1:500),

3. Anti-beta Actin (SP124) (Abcam ab8227 - Lot 1103556-1) (1:10,000),
4. Anti-mouse secondary (Abcam ab205719 Lot 1036603-15) (1:5,000),
5. Goat anti-rabbit secondary (Sigma A0545 Lot 069M4835V).(1:10,000),

Cytometric bead arrays (BD Biosciences) - as recommended by manufacturer
6. IFNA #560379 (lot 3117527),
7. TNF#560112 (lot 5013222),
8. IL12p70#558283 (lot 5121732),
9. IFNG # 558269 (lot 5031631).

Validation

Antibodies were validated by manufacturer as follows:
1.https://www.cellsignal.com/products/primary-antibodies/cd45-d3f8q-rabbit-monoclonal-antibody/70257
2.https://www.abcam.com/en-us/products/primary-antibodies/fibrillarin-antibody-38f3-nucleolar-marker-ab4566
3.https://www.abcam.com/en-us/products/primary-antibodies/beta-actin-antibody-loading-control-ab8227
4.https://www.abcam.com/en-us/products/secondary-antibodies/goat-mouse-igg-h-l-hrp-ab205719
5.https://www.sigmaaldrich.com/AU/en/product/sigma/a0545
6.https://www.bdbiosciences.com/en-au/products/reagents/immunoassay-reagents/cba/cba-kits/human-ifn-flex-set.560379
7.https://www.bdbiosciences.com/en-au/products/reagents/immunoassay-reagents/cba/cba-kits/human-tnf-flex-set.560112
8.https://www.bdbiosciences.com/en-au/products/reagents/immunoassay-reagents/cba/cba-kits/human-il-12p70-flex-set.558283
9.https://www.bdbiosciences.com/en-au/products/reagents/immunoassay-reagents/cba/cba-kits/human-ifn-flex-set.558269

Eukaryotic cell lines

Policy information about cell lines and Sex and Gender in Research

Cell line source(s)

HEK-Blue™ IFN-α/β Cells (Invivogen #hkb-ifnabv2-b) (used to make TLR7/8 mutant cells), 293XL-hTLR7 (#293xl-htlr7), 293XL-hTLR8 (293xl-htlr8), 293XL-hTLR9-HA (#293xl-htlr9ha), HEK-Blue™ hTLR3 (#hkb-htlr3) and HEK-Blue™ mTLR13 (#hkb-mtlr13) were purchased from Invivogen.
RAW ELAM macrophages were reported in PMID: 11686851
WT THP-1 cells were reported in PMID: 34057477
HEK-293T cells were published in PMID: 23722158
HipSci HPSI0114i-kolf_2 were from Sanger Institute
HeLa cells were purchased from ATCC #CCL-2
TLR4 KO iBMDMs were a gift from E. Latz

Authentication

None of the cells lines were authenticated.

Mycoplasma contamination

All cell lines tested negative for mycoplasma contamination using Mycostrip (Invivogen).

Commonly misidentified lines
(See ICLAC register)

HeLa cells - these were purchased from ATCC - and they were only used to obtain RNA as a modulator of TLR7/8 sensing

Animals and other research organisms

Policy information about studies involving animals; ARRIVE guidelines recommended for reporting animal research, and Sex and Gender in Research

Laboratory animals

Mice were housed in an specific pathogen free vivarium and fed normal chow ad libitum, mice experienced a 12 hour light/dark cycle with temperature ~22oC and humidity of ~40%.
1. 24x 8-week-old wild type C57Bl/6J female mice (Monash Animal Research Platform) were used for Aldara model experiments.
2. 3x 12-week-old wild type C57Bl/6J males (Monash Animal Research Platform) were used for Flt3L-derived DC preparations.
3. 12x 8-week-old female C57BL/6NCrl mice (ANU) were used for systemic R848 experiments.
4. 13x 8-week-old 129X1/SvJ female mice (JAX Lab:000691) were used for LNP experiments.
5. 3x 9-11-week old TLR7(WT/Kika)C57BL/6NCrl mutant female mice (ANU) were used for BMM purifications.

Wild animals

No wild animals were used in these studies.

Reporting on sex

TLR7 is expressed on Chromosome X and is linked to heightened auto-immunity in females; For this reason we selectively used female mice in all our experimentations.

Field-collected samples

No field collected samples were used in these studies.

Ethics oversight

1. LNP injection studies were approved by Institutional Animal Care and Use Committee reference IN020-08202020-27736.
2. Systemic R848 injections in C57BL/6NCrl were approved by Australian National University animal ethics, reference A2022/18.
3. Aldara driven skin inflammation studies were approved in advance by an Animal Ethics Committee at Monash Medical Centre (MMCB/2022/18 and MMCB/2023/19).
4. BMMs were collected from Tlr7Y264H C57BL/6NCrl mice (used under Australian National University animal ethics, reference A2021/29).
5. BMMs were collected from C57Bl/6J males for Flt3L-derived DC preparations (under Monash Medical Centre B Animal Ethics Committee reference MMCB/2024/30)

Note that full information on the approval of the study protocol must also be provided in the manuscript.

Plants

Seed stocks	NA
Novel plant genotypes	NA
Authentication	NA
